# An amebic liver abscess in a female child with successful recovery in a non-endemic area: a case report

**DOI:** 10.3389/fped.2025.1634486

**Published:** 2025-10-13

**Authors:** Jihang Jia, Qin Guo

**Affiliations:** ^1^ Department of Pediatrics, West China Second University Hospital, Sichuan University, Chengdu, China; ^2^ Key Laboratory of Birth Defects and Related Diseases of Women and Children, Sichuan University, Ministry of Education, Chengdu, China

**Keywords:** amebic liver abscess, liver abscess, *Entamoeba histolytica*, child, metronidazole

## Abstract

We report the case of a 2-year-and-11-month-old female child who presented with a 10-day history of recurrent fever and abdominal pain, accompanied by significantly elevated inflammatory markers. Initially, incomplete Kawasaki disease (IKD) was strongly suspected; however, the patient continued to experience recurrent high fever and abdominal pain despite treatment with intravenous immunoglobulin (IVIG) and antibiotics. Enhanced thoraco-abdominal computed tomography (CT) imaging revealed hepatomegaly and the presence of an abscess in the anterosuperior segment of the right lobe of the liver. The patient subsequently underwent a surgical intervention due to the progression of her symptoms, including worsening fever, abdominal pain, and the development of new-onset shortness of breath. The postoperative immunohistochemical analysis identified *Entamoeba histolytica* (*E. histolytica*) trophozoites, confirming the diagnosis of an amebic liver abscess (ALA). Following the diagnosis, the patient was treated with 10 days of oral metronidazole. A follow-up CT scan conducted 4 months later showed complete resolution of the liver abscess and normalization of liver function. While amebiasis is rare in pediatric populations, it should be considered in the differential diagnosis of liver abscesses, even in non-endemic regions.

## 1 Introduction

Amebiasis is an endemic disease that primarily occurs in tropical and low-income countries characterized by limited socioeconomic resources and poor sanitary conditions, such as India, West and East Africa, Mexico, and certain regions of Brazil (1). However, it can also affect travelers or immigrants returning from these endemic areas. The primary pathogen responsible for amebiasis is *Entamoeba histolytica* (*E. histolytica*), which is primarily transmitted via the fecal-oral route. A study conducted in China on the seroprevalence of *E. histolytica* reported overall positivity rates of 11.05% for crude antigens and 6.25% for the recombinant surface antigen C-Igl (2). The infection can lead to either asymptomatic colonization of the gastrointestinal tract or extraintestinal involvement, with organs such as the liver, lungs (3, 4), and brain (5) being commonly affected. An amebic liver abscess (ALA) is the most frequent extraintestinal manifestation of invasive amebiasis (6, 7). The abscess may grow considerably before clinical symptoms appear, and, in rare instances, can rupture into the pleural cavity, leading to acute, life-threatening respiratory distress. With early diagnosis and appropriate treatment, the prognosis for ALA is generally favorable.

## 2 Case presentation

A 2-year-and-11-month-old girl presented with a 10-day history of high fever, abdominal pain, diarrhea, and productive cough, without accompanying nausea, vomiting, or constipation. Her highest recorded temperature was 41°C, and she experienced distinct periumbilical pain during episodes of fever. No changes in the color or appearance of her stool were observed. Despite receiving antibiotics and intravenous immunoglobulin (IVIG, 1 g/kg) at a local hospital, her fever pattern and duration remained largely unchanged. The patient's condition had worsened 2 days prior to admission to our hospital's emergency department, with exacerbation of her fever, aggravated periumbilical pain, poor appetite, and vomiting. The patient was born prematurely at 28 weeks of gestation and currently resides in an urban environment with her family. A detailed medical history revealed no recent diarrhea or travel. No significant family medical history was reported.

Upon admission, the patient's physical examination revealed a temperature of 39.4°C, a respiratory rate of 32 breaths per minute, a heart rate of 180 beats per minute, and a blood pressure of 86/54 mmHg. No polymorphic or scarlatiniform rash was observed on the skin of her trunk or extremities. No jaundice was observed on her skin, sclerae, or mucous membranes. Exfoliation was noted on the tips of her fingers, and lymph nodes approximately 5 mm in size were palpable in her neck. Her lips were notably dry. The patient also displayed congested oral mucosa and a mild strawberry tongue. The cardiac and respiratory examinations were unremarkable. A physical examination revealed an abdominal circumference of 55 cm. The liver was palpable 3.5 cm below the xiphoid process and 3 cm below the right costal margin, with a soft consistency overall and no tenderness.

Blood tests revealed leukocytosis (20.5 × 10^9^/L), with neutrophils at 15.05 × 10^9^/L, lymphocytes at 3.42 × 10^9^/L, hemoglobin at 89 g/L, and platelets at 850 × 10^9^/L. The patient’s C-reactive protein (CRP) level was markedly elevated at 207.9 mg/L. Liver function tests showed alanine aminotransferase at 44 U/L, aspartate aminotransferase at 42 U/L, albumin at 31.3 g/L, total bilirubin at 52.5 μmol/L, conjugated bilirubin at 30.7 μmol/L, and unconjugated bilirubin at 21.8 μmol/L. Kidney function tests revealed urea at 2.34 mmol/L and creatinine at 23 μmol/L. In addition, ferritin was elevated at 947.10 ng/mL, serum amyloid A protein was 286.42 mg/L, and the erythrocyte sedimentation rate was 31 mm/h. The results from other serum tests are summarized in Table 1. Stool microscopy revealed no red blood cells, white blood cells, pus cells, phagocytes, fat globules, or fungi.

**Table 1:** Serum tests of the patient before admission and during hospitalization.

Test (range, unit)	Before admission	On admission	After IVIG	Before metronidazole	17 days after metronidazole
Leukocyte (3.6–13 × 10^9^/L)	14.8	20.5	26.2	5.3	4.8
Neutrophil (0.72–4.8 × 10^9^/L)	9.34	15.05	22.03	1.86	1.15
NEUT% (12.9–56.7)	63.1	73.4	84.1	35.1	23.9
Lymphocyte (1.44–7.98 × 10^9^/L)	3.82	3.42	2.41	2.55	2.92
LYMPH% (35.4–78.6)	25.8	16.7	9.2	48.1	60.8
Eosinophil (0–0.53 × 10^9^/L)	0.04	0.04	0.03	0.22	0.14
EOS% (0–6.4）	0.3	0.2	0.1	4.2	2.9
PLT (125–462 × 10^12^/L)	579	850	764	605	455
Hemoglobin (108–145 g/L)	93	89 g/L	58	100	123
CRP (0–8 mg/L)	173.7	207.9	287.7	11.6	2.3
Alanine aminotransferase (<49 U/L)	47	44	25	25	27
Aspartate aminotransferase (<40 U/L)	38	42 U/L	34	33	41
Total bilirubin (5–21 μmol/L)	8.6	52.5	37.9	9.4	10.5
Unconjugated bilirubin (<17 μmol/L)	8.6	21.8	11.6	4.9	10.5
Conjugated bilirubin (＜6.8 μmol/L)	0	30.7	26.3	4.5	0
Albumin (38–54 g/L)	29.8	31.3	25.5	50.6	46.5
urea (3.2–8.2 mmol/L)	2.34	3	3.1	6.39	4.97
Creatinine (17.3–54.6 μmol/L)	23	23	23	19	21
PCT (＜0.05 ng/mL)	2.4	NA	NA	0.15	NA
Ferritin (10–291 ng/mL)	NA	947.10	1,528.5	347.6	NA
SAA (0–10 mg/L)	286.42	NA	NA	NA	NA
Erythrocyte sedimentation rate (＜26 mm/h)	31	NA	NA	35	NA
D-dimer (＜0.55 mg/L)	NA	NA	7.9	1.05	NA
Blood ammonia (10–47 μmol/L)	NA	NA	78.5	NA	NA
CA125 (＜35 U/mL)	NA	146.6	NA	NA	NA
AFP (＜8.1 ng/mL)	NA	＜1.3	NA	NA	NA
CEA (＜2.5 ng/mL)	NA	＜0.5	NA	NA	NA
CA19–9 (＜30.9 U/mL)	NA	27.8	NA	NA	NA
ThCG (＜10 mIU/mL)	NA	＜2	NA	NA	NA

NA, not available; PLT, platelet; CRP, C-reactive protein; SAA, serum amyloid A protein; PCT, procalcitonin; AFP, alpha fetoprotein; CEA, carcinoembryonic antigen; ThCG, total human chorionic gonadotrophin.

**Figure 1:**
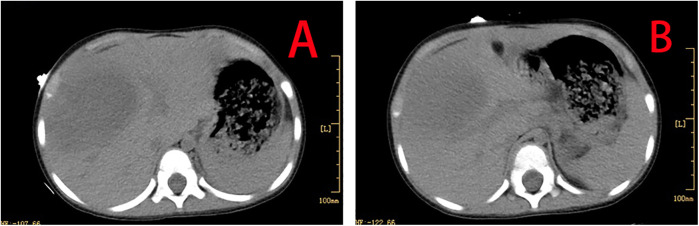
CT showing hepatomegaly, with extensive low-density areas in the left inner and right anterior lobes of the liver **(A,B)**.

The Coombs test was positive, while the rheumatism screening returned negative results. Both *Chlamydia pneumoniae* IgG and *Mycoplasma pneumoniae* total antibodies were positive. Blood and bone marrow cultures were obtained. Echocardiography revealed no abnormalities in the left and right coronary arteries or their branches. Non-enhanced abdominal computed tomography (CT) showed hepatomegaly, with extensive low-density areas in the left inner and right anterior lobes of the liver, the nature of which was unclear (Figures 1A,B).

A preliminary diagnosis of incomplete Kawasaki disease (IKD) was made upon admission, based on the patient's age, prolonged fever, clinical signs, elevated CRP (>30 mg/L), anemia, platelet count >450 × 10^9^/L after 7 days of fever, and albumin <30 g/L. Given the prior administration of IVIG (1 g/kg) at the local hospital, a second dose (1 g/kg) was promptly given. However, the patient continued to experience recurrent high fever, cough, polypnea, significant abdominal bloating, and abdominal pain post-IVIG. Follow-up blood tests revealed increased infectious markers (Table 1), and an abdominal examination indicated further hepatomegaly. A re-evaluation by echocardiography showed no abnormalities in the left or right coronary arteries or their branches. Considering the possibility of IVIG-resistant KD, a second dose of IVIG (2 g/kg) was administered. Concurrently, a diagnosis of sepsis was made based on the patient's clinical manifestations and markedly elevated leukocyte and CRP levels, prior to the availability of the blood and bone marrow cultures. Empirical therapy with intravenous ceftriaxone (50 mg/kg every 12 h) and vancomycin (10 mg/kg every 6 h) was initiated for a suspected bacterial infection. The blood cultures were negative 5 days postadmission, while the bone marrow culture grew *Staphylococcus epidermidis*, which is sensitive to vancomycin.

**Figure 2:**
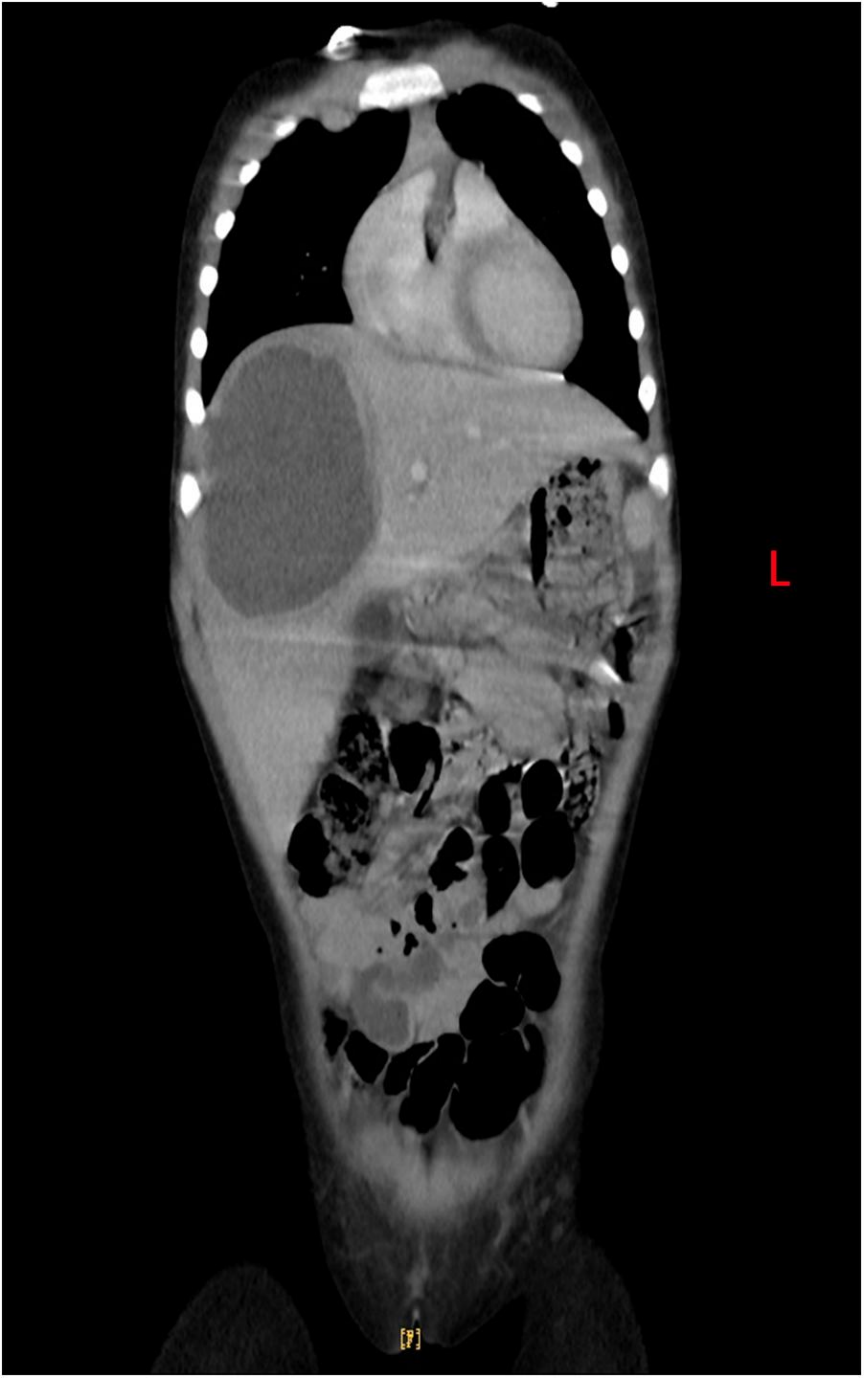
Enhanced thoraco-abdominal CT shows a suspected abscess in the anterosuperior segment of the right lobe of the liver.

At the same time, there was still high suspicion of a tumor or autoimmune disease. Serum tests for 17 autoantibodies were performed, all yielding negative results. Enhanced thoraco-abdominal CT imaging revealed inflammation in the upper lobe of the right lung and the lower lobes of both lungs, with consolidation in the posterior segment of the right upper lobe and partial thickening of the bilateral pleura. Abdominal imaging indicated hepatomegaly (measuring 8.4 cm × 6.3 cm × 8.5 cm) and a suspected abscess in the anterosuperior segment of the right lobe of the liver (Figure 2). Given the persistence of high fever, abdominal pain, worsening dyspnea, and pronounced hepatomegaly, the patient was referred for surgery prior to definitive characterization of the liver abscess. She underwent right partial hepatectomy, drainage of the liver abscess, and intestinal adhesiolysis. Bacterial cultures of the peritoneal drainage fluid were negative. However, chest radiography 4 days postsurgery revealed a right pleural effusion, which was managed with thoracic puncture and drainage for 3 days. Pleural fluid analysis showed nucleated cells at 1,200 × 10^6^/L, with 55% polymorphonuclear cells, 35% mononuclear cells, 1,490 × 10⁶/L red blood cells, total protein of 51 g/dL, albumin of 32.7 g/dL, lactate dehydrogenase of 795 IU/L, and adenosine deaminase of 11.9 IU/L, with the presence of pyocytes. Pleural fluid culture was negative. Immunohistochemical analysis of liver necrotic tissue confirmed the presence of amebic trophozoites, thereby establishing the diagnosis of ALA. The patient's fever resolved 12 days postoperation.

**Figure 3:**
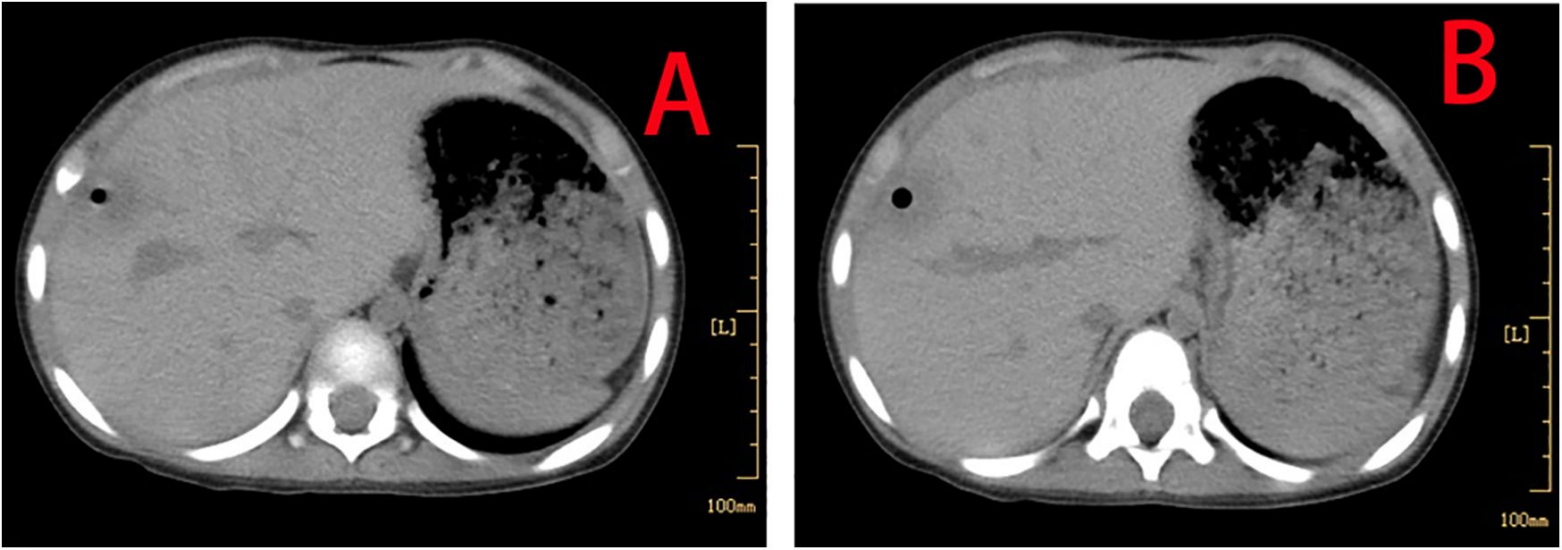
Enhanced thoraco-abdominal CT scans show a reduction in the size of the liver abscess **(A,B)**.

**Figure 4:**
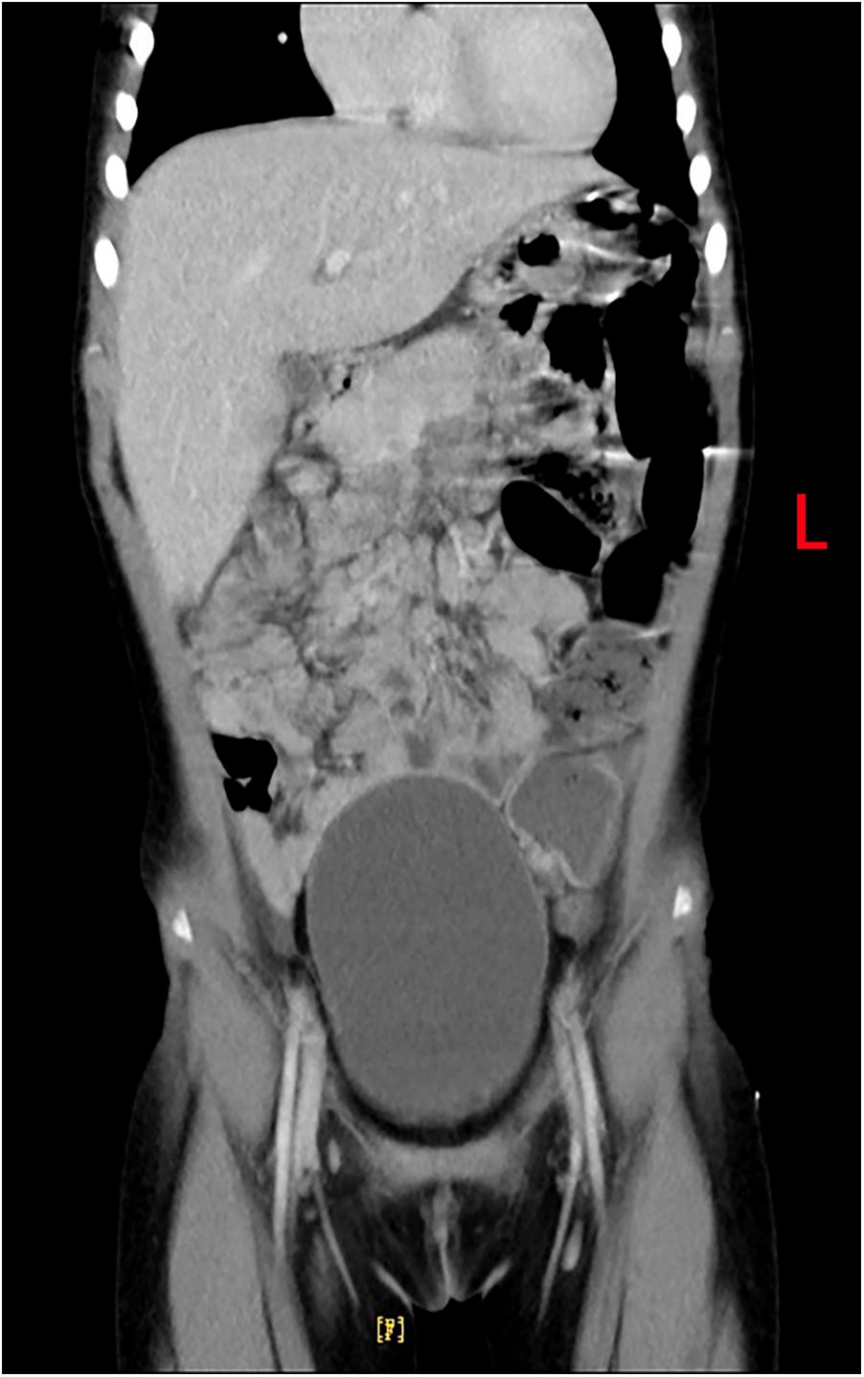
A follow-up CT scan 4 months later showing complete normalization of the liver.

The patient made a full recovery, with no further episodes of fever or abdominal pain, following a 10-day course of oral metronidazole (0.2 g orally, three times daily), in conjunction with continued vancomycin administration for a total of 40 days. Repeated infection markers and bone marrow cultures were negative. Enhanced thoraco-abdominal CT scans showed a significant reduction in the size of the liver abscess (Figures 3A,B), and abdominal ultrasound indicated gradual improvement of liver function prior to discharge. Remarkably, a follow-up CT scan performed 4 months later revealed complete resolution of the liver abscess and normalization of her liver architecture (Figure 4).

## 3 Discussion and literature review

An ALA is a rare condition in children, with reported cases occurring in infants as young as 20 days and extending to adolescents as old as 14.5 years (8, 9). There is a lack of data on the exact incidence of ALAs in children infected with *E. histolytica*. Three studies have reported the prevalence of ALAs in children, with rates of 1%–5%, 12%, and less than 1%, respectively (8, 10, 11), which is lower than the incidence in adults, which is reported to be 10%–50% in adults with invasive intestinal amebiasis (8). Historically, the mortality rate for an ALA was reported to be between 11% and 14% prior to 1984, but significant advancements in diagnosis and treatment have reduced it to less than 1% (12). Despite these improvements, delayed diagnosis and treatment continue to contribute to mortality today. A case in point is a 6-year-old Malay boy who died from acute respiratory distress after being misdiagnosed with perforated acute appendicitis and receiving delayed metronidazole administration (13). In the majority of cases, an ALA occurs without concurrent intestinal amebiasis (14). The clinical presentation typically includes fever, vomiting, right upper quadrant pain, and tender hepatomegaly. Diarrhea is observed in approximately 30% of pediatric cases, but detecting amebic trophozoites in stool examination remains extremely rare (15).

An ALA can lead to serious complications, including rupture into the pleural cavity, peritoneum, or pericardium, which can be fatal. For example, a 2-year-old child was not diagnosed with an ALA until the abscess ruptured into the pleural cavity, causing respiratory distress (16). Similarly, a case of massive pleural empyema secondary to a ruptured liver abscess has been reported in a male adolescent (17). A pleural effusion can usually be managed effectively with drainage and antimicrobial therapy (3). In our patient, the development of dyspnea, right pleurisy, and a pleural effusion during hospitalization was likely the result of complications secondary to the ALA.

The diagnosis of an ALA generally relies on a combination of clinical presentation, amebic serology, and radiological imaging (18). In some cases, a relevant epidemiological history, such as travel to endemic regions, can be helpful in making the diagnosis. Studies have shown that poor living conditions, consumption of nonpurified water, and eating food not prepared at home are major risk factors for amebiasis (19). Our patient, however, was a young toddler living in an urban area with adequate sanitation. No specific epidemiological risk factors or direct exposure to *E. histolytica* could be identified, leaving the true source of the infection unclear. This highlights the complexity of diagnosing an ALA in non-endemic regions where such infections are less anticipated.

Serological testing for antiamebic antibody is a useful method for diagnosing an ALA and other forms of extraintestinal amebiasis (20). In fact, serological tests are positive in approximately 90% of patients with an ALA (21). However, the utility of serology in endemic populations is limited, as it becomes challenging to distinguish between current, active infections, and past exposures or resolved infections. Therefore, while serological testing can be highly sensitive in specific clinical contexts, it must be interpreted with caution, particularly in settings where endemicity is high.

Ultrasound and CT are widely recognized as effective imaging modalities for detecting liver abscesses, with ultrasound demonstrating a sensitivity greater than 90% for identifying an ALA. Given its high sensitivity, ultrasound is often recommended as the initial imaging tool (12). However, in our case, a delay in performing the abdominal ultrasound proved to be a limitation, as it should ideally have been conducted upon admission to facilitate a timely diagnosis. Notably, on physical examination, the liver was non-tender and soft in consistency, which is atypical for an ALA. The absence of classic signs such as tenderness or a focal mass could be attributed to the deep-seated location of the abscess or its early stage, thereby posing a significant diagnostic challenge and potentially leading to a delay in ultrasound and diagnosis. While radiological imaging can identify the presence of a liver abscess, it cannot differentiate between an ALA and other space-occupying lesions, such as pyogenic liver abscesses. Although a CT scan helped identify the liver abscess in our patient, it could not determine the specific etiology of the infection, highlighting the need for additional diagnostic methods, such as serological testing or tissue biopsy, to confirm the causative organism.

While culture or microscopic examination of abscess aspirates can aid in diagnosing an ALA, detection of amebic trophozoites in pus aspirates is often challenging, as they are typically located in the abscess wall (20). Immunohistochemistry offers a higher specificity than traditional staining methods, such as hematoxylin and eosin (H&E) or periodic acid-Schiff (PAS) staining techniques. However, immunohistochemistry remains a relatively rare diagnostic tool for invasive amebiasis (22). In our patient's case, a surgical intervention and the availability of hepatic necrotic tissue ultimately allowed us to make a confirmatory diagnosis through immunohistochemistry.

Molecular diagnostic assays, such as conventional polymerase chain reaction (PCR), nested PCR, real-time PCR, multiplex PCR, and loop-mediated isothermal amplification (LAMP), have been widely used to detect *E. histolytica* DNA in biological specimens such as stool, tissue, or abscess aspirates. Recent studies suggest that nested multiplex PCR and SYBR Green real-time PCR assays provide accurate results for ALAs, outperforming conventional PCR (23). Moreover, the multiplex gastrointestinal (GI) PCR panel, which detects *E. histolytica* in stool and liver abscess fluid (off-label), led to a rapid diagnosis of an ALA in an adult (24). Despite these advancements, the widespread use of molecular diagnostics remains limited by cost and the need for specialized technical expertise, particularly in resource-limited settings.

The first-line treatment for extraintestinal amebiasis, including an ALA, is metronidazole or tinidazole, which effectively eliminates the invasive trophozoites. Oral or intravenous metronidazole is typically administered for 7–10 days, and oral tinidazole is an alternative with a 3–5-day course (15). Sequential treatment with intraluminal amebicides, such as paromomycin, iodoquinol/diiodohydroxyquinoline, or diloxanide furoate, is recommended following metronidazole therapy to clear luminal parasites and prevent relapse (15). Evidence supports the use of metronidazole alone for the treatment of an uncomplicated, solitary, right-lobe ALA up to 10 cm in size (25). In such cases, routine aspiration or drainage is not necessary (12). However, if the ALA is refractory to medical management, percutaneous or surgical aspiration and drainage may be necessary, with broad-spectrum antibiotics added if a pyogenic infection is suspected (12, 15). In our case, the patient received empirical antibiotics followed by a full course of metronidazole postsurgery. Unfortunately, due to issues with drug availability, subsequent treatment with paromomycin, iodoquinol, or diloxanide furoate could not be administered.

This case highlights two key lessons for pediatric clinicians. First, making a diagnosis based on a prolonged fever with elevated inflammatory markers in children can be inherently challenging. Our patient initially presented with symptoms that were consistent with IKD, which led to confusion at the time of admission. According to the *Guideline for the Diagnosis and Treatment of Incomplete Kawasaki Disease in Children in China* (26), the diagnosis of IKD appeared reasonable. IVIG was administered to prevent potential cardiovascular sequelae, despite a negative echocardiogram. After the first dose of IVIG, a diagnosis of IVIG-resistant KD was considered, prompting a second dose. However, the lack of response to IVIG and the eventual resolution of the symptoms following surgery and antibiotic treatment, pointed to an infectious etiology rather than KD. This case highlights that in cases of prolonged fever where KD is suspected, and there is a poor response to IVIG, clinicians should broaden their differential diagnosis to consider potential infectious causes and systematically screen for infection foci. Second, this case underscores the importance of considering uncommon pathogens when standard antibacterial treatment fails. While pyogenic liver abscesses account for the majority (approximately 80%) of liver abscess cases, both amebic and fungal liver abscesses have been reported with increasing frequency in recent years (12). This case further highlights that clinicians should remain vigilant for rare pathogens, particularly when the clinical response to the initial therapy is inadequate.

## 4 Conclusion

The diagnosis of ALAs in the pediatric population presents significant challenges, particularly in non-endemic regions. This case illustrates that it is crucial to consider an ALA in pediatric patients presenting with a liver abscess regardless of their geographical background. Initially misdiagnosed as IKD, this febrile case highlights the importance of expanding the differential diagnosis when encountering persistent fever and elevated inflammatory markers. It also emphasizes the need for clinicians to consider further diagnostic workups, including imaging and histopathological analysis, to ensure accurate and timely diagnosis when an ALA is a possibility, given its ability to mimic other conditions such as KD.

## Data Availability

The original contributions presented in the study are included in the article/Supplementary Material, further inquiries can be directed to the corresponding author.
